# Lose sleep, lose score? Interpreting computerized neurocognitive testing after concussion

**DOI:** 10.1007/s44470-026-00107-6

**Published:** 2026-07-07

**Authors:** Amir Sharafkhaneh, J. Kent Werner, Ahmed BaHammam

**Affiliations:** 1 Department of Medicine, Baylor College of Medicine, Houston, TX USA; 2 Department of Neurology and Rehabilitative Medicine, Uniformed Services University, Bethesda, MD USA; 3 Department of Medicine, College of Medicine, University Sleep Disorders Center, The Strategic Technologies Program of the National Plan for Sciences and Technology and Innovation in the Kingdom of Saudi Arabia; King Saud University-Medical City (KSU-MC), King Saud University, Riyadh, Saudi Arabia; 4 Sleep Disorders & Research Center, Baylor College of Medicine, Michael E. DeBakey VA Medical Center, Houston, TX USA

**Keywords:** Traumatic brain injury, Sleep duration, Sleep disturbance, Cognition, Cognitive dysfunction

Traumatic brain injury is prevalent, resulting from various injury settings, and results in persistent cognitive changes. Traumatic brain injury (TBI) affects processing speed, attention, memory, and executive function. The PROTECT-TBI cohort of 15,764 participants showed a dose- and severity-dependent association with attention and executive function, demonstrating half the processing speed and working memory of controls [1].

How do these cognitive adversities present clinically? One patient reported, “It’s like my brain has a hole, and things tend to fall out.” [2] In the context of physicians’ day-to-day interactions with their patients, these changes may appear as failures to understand and follow instructions (processing speed), not remembering prior test results and instructions (memory), not being able to organize their lives around their responsibilities and taking care of themselves (executive function), or driving while sleepy (decision making).

The relationship between TBI symptoms and sleep disorders is bidirectional. Up to 50% of patients with TBI develop one or more sleep disorders, including insomnia, hypersomnia, restless legs syndrome, and circadian rhythm disorder. In turn, sleep disorders are associated with TBI symptom exacerbation [3]. Mechanistic work indicates disrupted glymphatic clearance after TBI, with aquaporin-4 mislocalization and reduced perivascular outflow that may compound axonal injury during fragmented sleep [4].

So, how does sleep influence TBI’s effects on cognition? A study from the Million Veteran Program (MVP) evaluated the association of sleep with the relationship between TBI and poor cognition [5]. The study, which employed a subjective cognition scale (MOS-Cog-R) in a post-deployment cohort of Veterans, showed that reduced sleep duration and greater sleep disturbance are associated with poorer subjective cognitive symptoms. In contrast to the MVP, Aderman and colleagues, using an objective tool, “computerized neurocognitive testing (CNT),” proposed that sleep duration measured the night before testing exerts a measurable influence on test performance among US service academy cadets and midshipmen in their study recently published in the *Journal of Clinical Sleep Medicine* [6].

Their analysis across large baseline and post-injury cohorts demonstrates that even modest differences in sleep duration contribute to detectable changes in verbal memory, visual memory, visual motor speed, and reaction time scores on the Immediate Post-Concussion Assessment and Cognitive Testing (ImPACT) battery, as well as selected Automated Neuropsychological Assessment Metrics (ANAM) outcomes, particularly at early clinical time points in concussion recovery.

In the present study by Aderman and colleagues, shorter sleep duration was associated with poorer performance on core ImPACT metrics across multiple assessment points, including baseline and the initial post-injury evaluation [6]. These findings align with evidence that sleep disruption directly impairs attention, memory, and processing speed, the very domains that computerized concussion tools target. A complementary observation comes from a study that analyzed more than 25,000 ImPACT records and reported that hours of sleep barely altered baseline composites yet meaningfully shifted post-injury verbal memory, visual memory, impulse control, and symptom severity [7]. The pattern suggests that the uninjured brain compensates for a single short night, whereas the injured brain has thinner reserves and amplifies the cognitive cost of the same sleep loss. Pre-injury sleep complaints may set this stage: athletes with pre-existing sleep difficulties showed slower reaction time and higher symptom burden up to 2 weeks after concussion compared with those without [8].

One of the most clinically actionable aspects of the Aderman study is its identification of sleep as a modifiable and easily assessed contributor to CNT variability. Sleep duration is typically neither standardized nor routinely recorded in many clinical settings. However, the current findings, reinforced by evidence from athletic and pediatric concussion populations, suggest that failing to document sleep can obscure interpretation of cognitive change over time. Short sleep can mimic or amplify concussion-related deficits, potentially leading to unnecessary delays in return-to-activity or return-to-duty decisions. Conversely, adequate sleep may temporarily mask subtle deficits.

Beyond ImPACT, the sleep effect was smaller on the ANAM composites in the Aderman analysis. It is genuinely unclear why. The two batteries may differ in how much they react to a short night, but practice effects, ceiling effects on ANAM, and the sample size needed to detect a small signal are equally plausible. ImPACT memory and reaction time look particularly susceptible to short sleep; the ANAM result should not be read as evidence that sleep is irrelevant to that battery.

Sleep duration should be documented routinely before every CNT, and abnormal post-injury scores should be interpreted cautiously when the prior-night’s sleep was less than 6 h. A repeat assessment after a normal night can help separate signal from noise, especially before duty or activity clearance. A promising technological development is pervasive use of consumer wearables that could provide objective measures of sleep [9]. Active screening for insomnia, hypersomnia, and sleep-disordered breathing across recovery, with treatment when indicated, may improve both cognitive functioning and overall recovery trajectory.

Caution is in order. Partial *r*^2^ values under 0.05 are statistically real but clinically modest, and whether an added hour of sleep actually shortens return-to-activity has not been tested. Sleep was scored from a single self-report item collected the night before, so sleep quality, chronic restriction, and a shift in circadian phase were never captured. We do not know, from the data, if sleep disturbance with no change in sleep duration will have similar or differing effects. Concussion can worsen sleep, and poor sleep can slow recovery; an observational design cannot tell those two directions apart.

TBI happens at all ages. Those who were tested in this study are young, otherwise healthy adults. Further data on older populations with various comorbid conditions are needed to extend these findings. Sleep-disordered breathing was not assessed, and the study did not record medication, caffeine, alcohol, or stimulant use, although each can alter CNT performance.

In conclusion, the Aderman study confirms that sleep duration affects CNT performance both before and after concussion. Together with the broader sleep and TBI literature, the message for clinical practice is straightforward: prior-night sleep should be recorded on every CNT, abnormal post-injury scores should be reinterpreted when sleep was short, and ongoing sleep complaints should be evaluated and treated. Sleep is modifiable, biologically relevant, and overlooking it leaves an avoidable gap in concussion assessment.

## Data Availability

There is no data with this commentary.

## Declarations

### AI disclaimer

The AI was used for formatting and improvement of wording and grammar.
